# The role of neural correlations in a decision-making task

**DOI:** 10.1186/1471-2202-14-S1-O10

**Published:** 2013-07-08

**Authors:** Federico Carnevale, Víctor de Lafuente, Ranulfo Romo, Néstor Parga

**Affiliations:** 1 Departmento de Física Teórica, Universidad Autónoma de Madrid, Cantoblanco 28049, Madrid, Spain; 2 Instituto de Neurobiología, Universidad Nacional Autónoma de México, Querétaro 76230, México; 3 El Colegio Nacional, 06020 México DF, México; 4 Instituto de Fisiología Celular, Universidad Nacional Autónoma de México, 04510 México DF, México

## 

Simultaneous recordings of the firing activity of pairs of cortical neurons have shown that spike-count correlation coefficients (CCs) cover a wide range of values. According to recent theoretical and experimental work [1,2] recurrent cortical networks decorrelate neural activity producing very low CCs. However little is known about the origin of correlations and data analysis based on recordings of cortical activity of awake, behaving animals performing non-trivial tasks are scarce. In this study, we aim to understand the role of neural correlations in perceptual decision-making tasks and its relationship with the covariation between neural firing activity and behavior.

We examine spike-count correlations obtained from pairs of simultaneously recorded premotor cortex (PC) neurons while trained monkeys performed a vibrotactile detection task in which the stimulus was often absent or weak, and the time of its application was variable [3,4]. By analyzing firing rates and correlated variability we show that behavioral outcomes are crucially affected by the state of cortical networks before stimulus onset times.

Our results suggest that sensory detection is partly due to a purely internal signal whereas the stimulus, if finally applied, adds a contribution to this initial processing later on [5]. Noise correlations can be weak; their smallest values are attained at the end of the delay period of the task. Importantly, we found that small CCs are compatible with high firing rates. Although the firing rate in hit trials is higher than in correct rejections, the distributions of CCs over the population of pairs are similar, presenting mean values of 0.06. Moreover, the CCs do not covary with the geometrical mean of the firing rate of the pair.

The observation that single neurons covary with the subject's response (characterized by the choice probability index, CP) is usually explained by the existence of variability correlations among the cells in a neuronal population [6]. Here we show a simple approximate expression that explicitly relates the population-averaged CP index and the CCs. This expression shows that the CP index is different from 0.5 when CCs evaluated using all trials differ from choice-conditioned correlations. Neurons could covary significantly with behavior even if the latter are very small. Thus, we show that there is no contradiction between the correlated activity required for the non-chance CP index and the small correlations produced by decorrelation in recurrent networks.

Although the CP index is useful to study the role of single neurons in decision-making tasks, in reality, the decision is formed through the coordinated action of several pools of neurons. Hence, the relevant quantities to investigate the elaboration of the choice are population variables combining the activity of several pools. By extending the notion of CP from single neurons to neural pools, we defined the CP_N _index to quantify the amount of covariation with behavior of arbitrary linear combinations of oppositely-tuned neural pools. We found that pools of PC neurons exhibiting persistent activity become fully correlated with the subject's choice soon after stimulus onset and during the entire delay period of the task.
